# Hidden biases in germline structural variant detection

**DOI:** 10.1186/s13059-021-02558-x

**Published:** 2021-12-20

**Authors:** Michael M. Khayat, Sayed Mohammad Ebrahim Sahraeian, Samantha Zarate, Andrew Carroll, Huixiao Hong, Bohu Pan, Leming Shi, Richard A. Gibbs, Marghoob Mohiyuddin, Yuanting Zheng, Fritz J. Sedlazeck

**Affiliations:** 1 Human Genome Sequencing Center, Baylor College of Medicine, Houston, TX USA; 2 Department of Molecular and Human Genetics, Baylor College of Medicine, Houston, TX USA; 3 Roche Sequencing Solutions, Santa Clara, CA USA; 4 DNAnexus, Mountain View, CA USA; 5 National Center for Toxicological Research, Food and Drug Administration, Jefferson, AR USA; 6 State Key Laboratory of Genetic Engineering, Human Phenome Institute, School of Life Sciences and Shanghai Cancer Center, Fudan University, Shanghai, China; 7 Institute of Thoracic Oncology, Fudan University, Shanghai, China

**Keywords:** Next-generation sequencing, Structural variations, Genomic variability

## Abstract

**Background:**

Genomic structural variations (SV) are important determinants of genotypic and phenotypic changes in many organisms. However, the detection of SV from next-generation sequencing data remains challenging.

**Results:**

In this study, DNA from a Chinese family quartet is sequenced at three different sequencing centers in triplicate. A total of 288 derivative data sets are generated utilizing different analysis pipelines and compared to identify sources of analytical variability. Mapping methods provide the major contribution to variability, followed by sequencing centers and replicates. Interestingly, SV supported by only one center or replicate often represent true positives with 47.02% and 45.44% overlapping the long-read SV call set, respectively. This is consistent with an overall higher false negative rate for SV calling in centers and replicates compared to mappers (15.72%). Finally, we observe that the SV calling variability also persists in a genotyping approach, indicating the impact of the underlying sequencing and preparation approaches.

**Conclusions:**

This study provides the first detailed insights into the sources of variability in SV identification from next-generation sequencing and highlights remaining challenges in SV calling for large cohorts. We further give recommendations on how to reduce SV calling variability and the choice of alignment methodology.

**Supplementary Information:**

The online version contains supplementary material available at 10.1186/s13059-021-02558-x.

## Background

Structural variations (SVs) are an important class of genomic variation that are often defined as being 50 base pairs (bp) or larger. SVs are categorized as deletions, duplications, inversions, insertions, translocations, and complex rearrangements [1–3]. They have a profound impact on evolution, diseases including neurological disorders [4–6], Mendelian disorders [7, 8], and cancer [9, 10]. Thus, the reliable detection of SVs is becoming increasingly valuable for both research and clinical applications.

The identification of SV from next-generation sequencing (NGS) data is still not routine and suffers from multiple issues [1, 2, 11]. Several ongoing efforts aim to improve the reliability of SV calling by developing new benchmark sets, based on the application of multiple sequencing technologies [12]. The Genome in a Bottle (GIAB) consortium recently assembled a highly curated list of SVs for one trio and the Human Genome Structural Variation (HGSV) consortium has released highly accurate sets of SVs across 15 genomes [13–15]. These studies provided highly accurate SV sets for a limited number of trios [12, 14]. The majority of genomic studies rely on short-read-Illumina DNA sequencing [16] and leverage GIAB and HGSV data to provide important insights into the performance of methods used to analyze SVs from short-read data [17]. Prior studies do not, however, address the low reproducibility of SV calls from NGS data. In contrast, studies of low reproducibility of single-nucleotide variants (SNVs) [18, 19] have revealed the importance of standardized workflows and protocols to improve reproducibility; these are now used or prepared for analysis of data from large consortia (e.g., Trans-Omics for Precision Medicine (TOPMed), HGSV, All of US, etc.). The standardized workflows can reduce the technical sources of low reproducibility and substantially improve the ability to study biological variation, including novel sequences in samples from diverse populations. However, these steps are likely insufficient for SV as they may be impacted by other challenges.

**Fig. 1:**
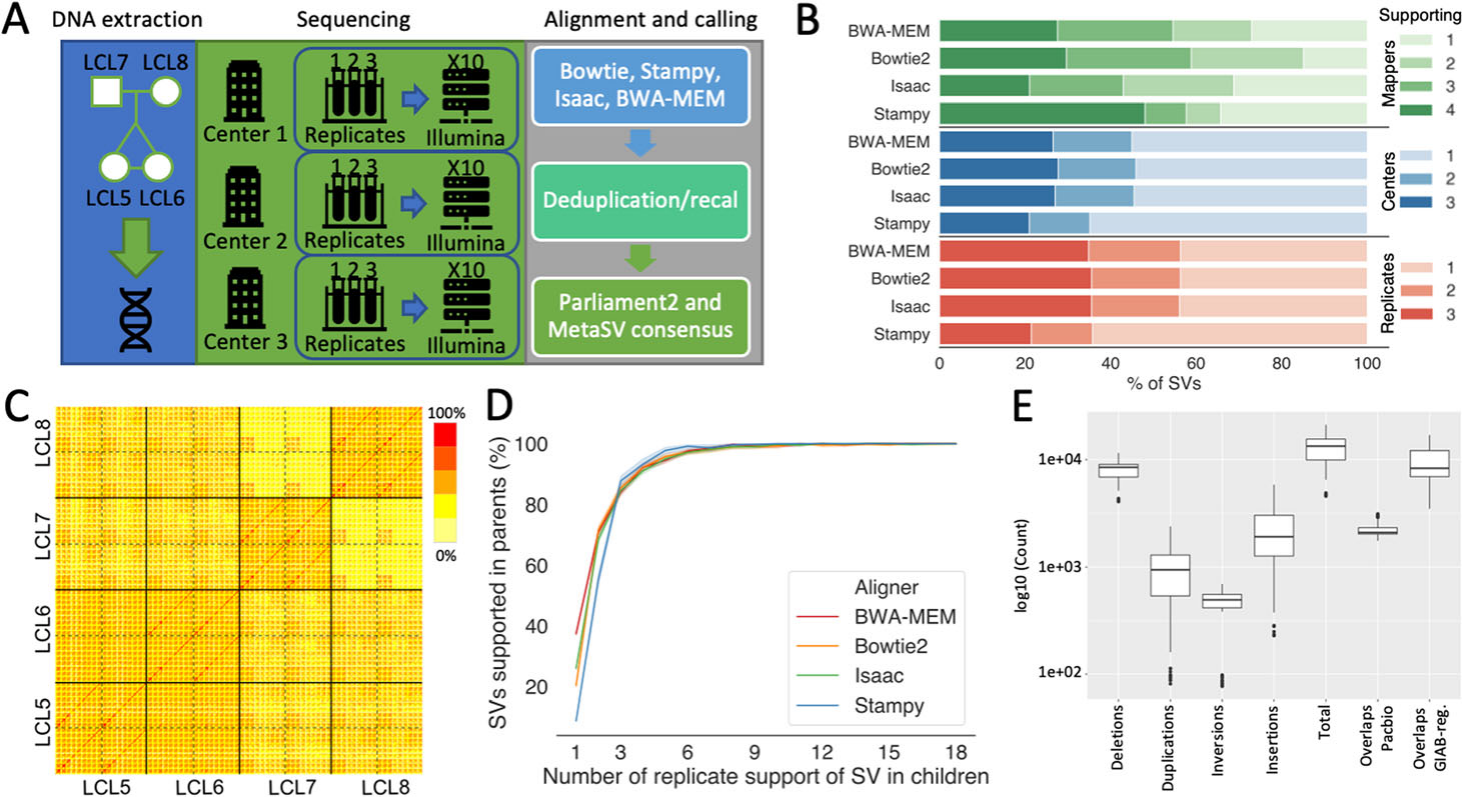
Overall study design and variability. **A** Sequencing and analysis overview of the Chinese quartet. The samples were sequenced in three replicates at three different centers. The files were then analyzed by four different mappers including base quality score recalibration (recal) and duplicate read marking (dedup). **B** Overlap and level of support between the different centers, replicates, and SV mappers. **C** Heatmap of the percent overlap between the different samples (red = high, yellow = low). **D** Assessing Mendelian consistency in identical twins compared to parents by mapper. The *x*-axis shows SVs that were called in replicates from identical twins (3 replicates x 3 centers x 2 twins = 18 replicates). The *y*-axis shows the percentage of SVs that were called in at least one of the replicates in parents (LCL7 and LCL8). **E** Distribution of SV events along the differently generated sample files for LCL5

To assess the impact of different sequencing centers, replicates, mappers, and platforms on the SV calling performance for NGS, we studied a Chinese family quartet from the Sequencing Quality Control Phase II (SEQC2) study [20]. The family consists of the biological parents (LCL7 & LCL8) and two monozygotic twins (LCL5 & LCL6) that were sequenced as three replicates at three different sequencing centers resulting in 288 SV call sets, respectively [20] (Fig. 1A). The in-depth analysis of LCL5 and the comparisons to family members demonstrated the contribution of different factors to SV call set variability. Specifically, we discerned which SVs resulted from false positives (falsely detected) or false negatives (missed in SV sample call sets) based on the variability of the sequencing process (sequencing centers and replicates) and the subsequent analyses (e.g., mapping methods: Isaac [21], Stampy [22], BWA-MEM [23], and Bowtie2 [24]). We assessed the accuracy and variability of the NGS call sets through comparing them to matched Pacific Biosciences (PacBio) Continuous Long Read (CLR)-based SV calls. Based on these analyses, we detailed the characteristics of the SVs that were introduced and potentially missed due to certain methodologies. We further provided mechanisms on how to control for these variabilities.

## Results

### Overall identification of variability

Each of the four family members was sequenced using NGS Illumina technologies over three replicates and across three different sequencing centers. Each resulting data set was processed using four mappers and subsequently marked for PCR duplicated reads (dedup) as well as base quality score recalibration (recal) (see the “Methods” section). This generated 72 data points per individual and a total of 288 for the entire family. We obtained a stringent set of SVs for each data point utilizing combined calls from MetaSV [25] and Parliament2 [26] to avoid generating high variability from individual SV callers (Fig. 1A, Methods for details) [17]. Overall, the call sets showed higher variability between the parents (LCL7 and LCL8) as expected than within the identical twins (LCL5 and LCL6) (Fig. 1C, D). On average, we identified 12,723.36 SVs across all 72 SV call sets from LCL5. The majority of these were deletions (7990.26 SVs) followed by insertions (2330.22 SVs) and duplications (997.38 SVs). Figure 1E shows a summary of the number, variability, and different types of SVs detected (see Additional file 1: Table S1 for details) across LCL5. The SV distribution followed the expected number and type of SVs observable by short reads (Fig. 1E) [12]. On average, 9479.35 SVs overlapped with the GIAB consortium high-confidence regions (see the “Methods” section). As expected, most SVs were in intergenic regions followed by intragenic regions (Additional file 2: Table S2).

For this study, we also produced a SV call set using long-read sequencing (see the “Methods” section) to examine the quality of the short-read SV calls; previous reports demonstrate that long-read sequencing approaches often improves the sensitivity and false positive rates compared to NGS [12, 14, 27, 28]. For PacBio LCL5, we detected 15,171 SVs of which 7465 (49.21%) were insertions and 6412 (42.26%) were deletions. This follows the expected SV type distribution that has been previously observed by GIAB [12]. For LCL5, 7,734 (50.98%) SV calls from Illumina SV call sets overlapped with the PacBio SV call set. The vast majority of missed SV calls in the Illumina call sets were insertions (4308) followed by deletions (1863). For LCL5, on average per Illumina call set, we only observed 2279.99 overlapping SVs, where the majority were deletions. These data further demonstrated the large variability across the Illumina call sets.

To investigate this variability further, we mapped the pairwise overlap of each data set across the entire family. Figure 1C shows this overlap across a heatmap. While the general trend is encouraging — we observe more similarity between the siblings (LCL5+LCL6) compared to the parents (LCL7+LCL8) — it becomes apparent that there are methodological differences within each sample that appear to be systematic.

### Short-read SV variability detection

**Fig. 2:**
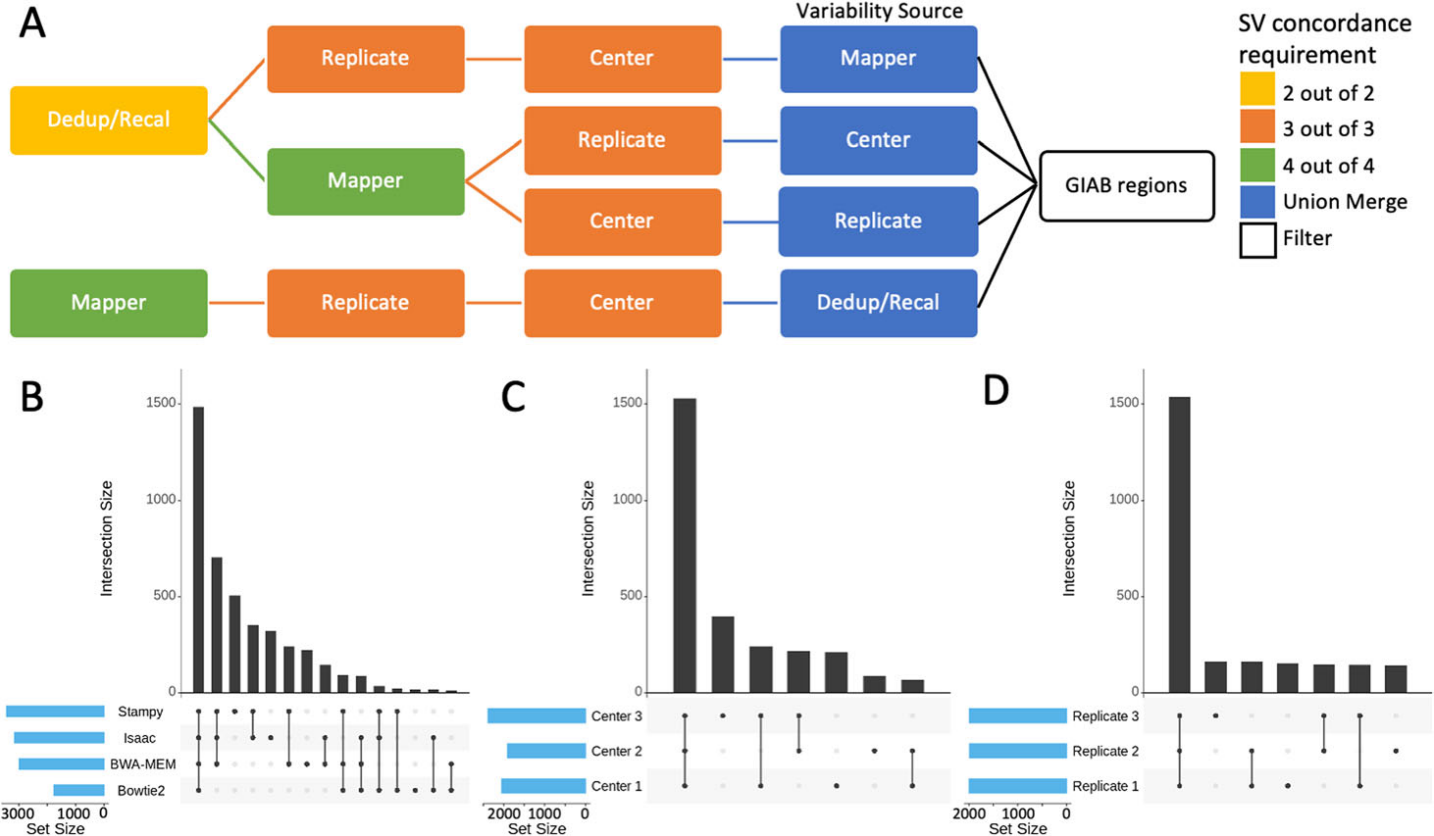
Analysis strategy and comparisons of mapper, center, and replicate SVs. **A** Analysis strategy for examining variability attributed to different factors including centers, replicates, mappers, and dedup/recal. To examine variability from each factor independently, SVs had to be concordant between the call sets of the other factors to pass for downstream analysis (e.g., to examine variable SVs attributed to mappers, SVs must be filtered to only include SV calls present in all replicates, centers, and dedup/recal). **B** Comparison of variability due to different SV mapping methods in LCL5. **C** Comparison of variability due to different centers in LCL5. **D** Comparison of variability across different replicates in LCL5

Investigating variability between Illumina SV call sets required preliminary analyses of SV prediction across short-read SV mappers, sequencing centers, and replicates. On average, 26.86% of the SV were supported by 1 out of 4 mappers. More specifically, mapper calls supported by one center represented 57.14% of SV calls and mapper calls supported by one replicate represented 48.88% of SV calls (Fig. 1B). These variabilities highlighted the contribution of different factors (mappers, sequencing centers, replicates, and dedup/recal) to variability in SV calling. This is also apparent when considering the family structure as singleton (called in one replicate) SV calls in the twins are often (23.13% on average) not supported by the call sets of the parents (Fig. 1D). This reaffirmed a general high variability between the different centers, replicates, and approaches. To investigate each source of variability independently, we stratified for other analytically accessible causes of variability and compared the outcomes across the family. Figure 2A illustrates the use of this analytical strategy in investigating the different sources of variability in this data set.. For example, concordant SV calls between centers, replicates, and dedup/recall SV call sets enable examining variability between mappers (see “Methods” section for further detail). Across strategies, we observed that most singleton SVs were deletions, and a minority were insertions; this was consistent with the pattern observed in the total number of SVs per stratification strategy (Additional file 3: Table S3). The majority of SVs including singleton SVs are 100–1000bp in size followed by 1000–10,000bp (Additional file 4: Table S4). Moreover, mapper variability was enriched with insertions (13.23%) compared to other strategies (less than 2%), which highlights the deficiencies in identifying insertions by mappers.

### Variability due to mapping methods

First, we investigated the contribution of different mapping methods (BWA-MEM, Bowtie2, Isaac, and Stampy) to the variability of SV calling after stratifying for other sources (e.g., centers, replicates, and dedup/recal) of variability (Fig. 2A). The variability attributed to different mappers per sample was the largest in comparison to other sources of variability (Additional file 5: Table S5). This was exemplified by Bowtie2, which did not report split reads leading to the smallest number of SVs identified (Fig. 2B). Generally, most SVs were supported by one or two mappers (Fig. 2B). Stampy showed the largest number of SVs per sample followed by Isaac; accordingly, both Stampy and Isaac showed the largest overlap with other mapping methods (Fig. 2B). Bowtie2, BWA-MEM, Stampy, and Isaac SVs overlapped the call set of at least one other method on average at 99.23%, 92.76%, 89.95%, and 85.19%, respectively.

Next, we investigated LCL5 and identified 4257 SVs across mappers after stratifying for other variabilities (Fig. 2B). SVs supported by one mapper (i.e., singletons) represented 25.02% (1065) of the total SVs and most of them (903 SVs, 84.79%) did not overlap with the PacBio call set. This high rate of non-overlapping SVs with the PacBio set indicated a high false positive rate for the singletons across the different mappers. For the non-singleton SVs (3192 SVs, 74.98%), we observed a substantial increase in the overlap with the PacBio call set to 60.35% (1926 SVs). Thus, the majority of the SV calls are likely true positives. Discrepancies between the call sets demonstrated that not all mappers capture all SVs, leading to increased variability between the SV call sets. Moreover, SVs were likely missed due to the differences between the SV mapping methods at that specific location. Thus, SVs supported by one (15.21% overlap), two (31.81% overlap), three (43.09% overlap), or four (87.13% overlap) mappers overlapped the PacBio call set at an increasing rate, respectively.

**Fig. 3:**
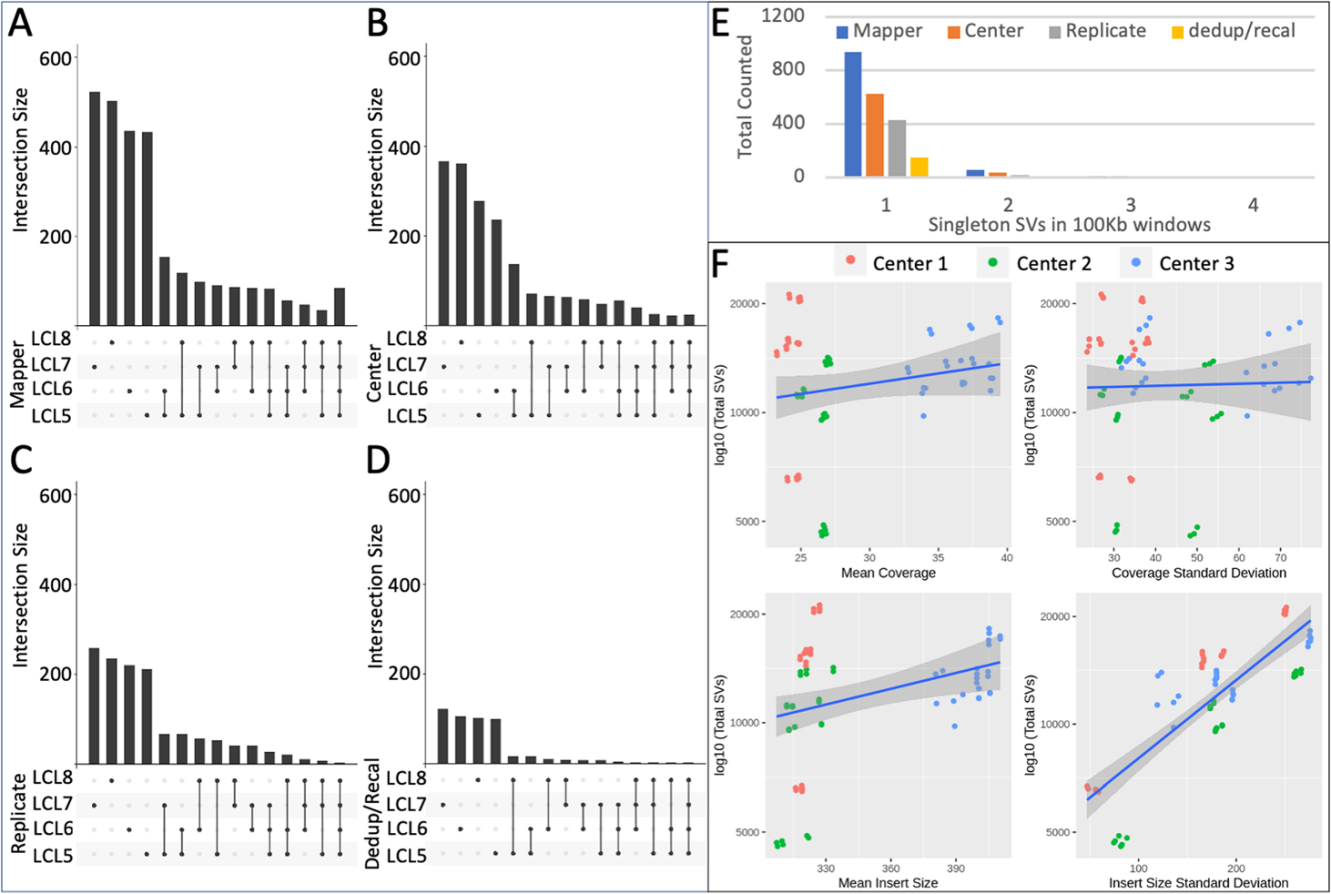
Comparison of singleton (unique) SV calls across family members for SV mapper (**A**), sequencing center (**B**), replicate (**C**), and dedup/recal (**D**) strategies. **E** Examining clustering of singleton SV in LCL5 across 100kbp windows genome-wide for each of the variability sources. **F** Scatter plots of total SVs identified per sample compared to mean and standard deviation of coverage and insert size, respectively. Each dot represents an SV call set with red representing Center 1, green representing Center 2, and blue representing Center 3

We observed that 59.25% of the SVs (631 SVs) supported by one mapper for LCL5 overlapped with mapper singletons from other family members (Fig. 3A). Both family overlapping and non-overlapping SVs were highly discordant with PacBio at 12.68% and 19.35%, respectively (Additional file 6: Table S6). This was consistent with a recurrent noise pattern pointing to false positive SV calls based on different SV mapping methods.

### Variability due to different sequencing centers

The second largest contribution to variability was observed across sequencing centers (Fig. 2C). This was likely due to differences in coverage and sequencing library preparation including insert size variability. We observed that Center 1 showed the lowest average coverage (24.37x) followed by Center 2 (26.33x) and Center 3 (36.33x) (Fig. 3F). The insert size also varied on average with Center 1 (321.55 bp), Center 2 (318.53 bp), and Center 3 (399.12 bp) (Fig. 3F). Overall, we identified a slightly positive correlation in the number of detectable SV with an increase in coverage or insert size. Using Pearson correlation, we observed only a slight correlation between total number of SVs and the insert size that was statistically significant (cor=0.26, *p* value=0.028). This was likely because the increase in insert size leads to a larger span of the read pairs, and thus, a higher likelihood that they spanned a breakpoint. Accordingly, more pairs supported a certain SV compared to a smaller insert size library. We observed no statistically significant correlation between total SVs and mean coverage (cor=0.12, *p* value=0.33). This is likely the result of standardized workflows between the labs as described in the “Methods” section.

Next, starting with LCL5, we stratified for other sources of variability as described in our analysis strategy and examined variability across centers (Fig. 2A). Post-stratification, we obtained 2750 SVs for the three sequencing centers (Fig. 2C). We observed that a majority of the center variability was attributed to SVs that were supported by one sequencing center (i.e., singletons) with 696 (25.31%) SVs. Center 3 supported the highest number of singleton SV calls per center followed by sequencing Centers 1 and 2. Interestingly, 45.12% (314 SVs) of these singleton SVs overlapped with the PacBio SV call set. Thus, they were likely true positives and indicated that these singletons represented false negatives based on other centers. The variability in coverage, insert size, and library preparation, discussed previously, contributed to the differences observed between the centers (Fig. 3F). Overlapping SVs between two of the three centers resulted in 525 (19.09%) SVs of which we observed an even higher rate of 59.23% (311) SVs overlapping with the PacBio-based SV calls. The increase in false negatives and the decrease in false positives indicated an expected higher rate of SVs missed in individual centers. After this stratification strategy, 1529 SVs (55.6%) overlapped between all three centers and were concordant with the PacBio SV call set (87.25% or 1334 SVs). Overall, and compared to mappers, center variability SVs were concordant at a much higher rate with the PacBio data set highlighting that these were not false positives as has often been believed. Rather, individual centers captured true positives representing false negatives for the other centers.

Next, we investigated if these singleton SVs per center were similar across the family members (Fig. 3B). For LCL5, 59.91% (417 SVs) of singleton SVs overlapped with LCL6, LCL7, and/or LCL8 singletons. Of these overlapping SVs, 47.72% (199 SVs) overlapped with the PacBio SV call set (Additional file 6: Table S6). This indicated a high recurrent incidence of missed SV calls (false negatives) in the call sets of multiple centers even across related samples (Additional file 7: Fig. S1). Singleton SVs that are non-overlapping with other family members amounted to 40.09% (279 SV) for LCL5 across the centers. Of these SVs, 41.94% (117 SVs) overlapped the PacBio call set and were likely false negatives.

### Other minor variability impacting SV detection

Subsequently, we analyzed variability attributed to differences between replicates per sample (Fig. 2D). Starting again with LCL5, we stratified for other causes of variability to obtain an SV set representing replicate variability (Fig. 2A). This resulted in 2453 SVs including 18.79% (461) supported by a single replicate, 18.51% (454 SVs) agreeing between two replicates, and 62.70% (1538 SVs) agreeing among all three. Over half (54.88%) of the singleton SV calls in LCL5 did not overlap with the PacBio SV call set and were likely false positive calls. The concordance with PacBio increased as supporting replicates increased from two replicates (64.10%) to three (87.26%). This indicated a decrease in false positives and an increase in false negatives for SVs supported by two replicates. Overall, we observed similar trends for other samples (Additional file 5: Table S5).

Next, we investigated the overlap among family members by aggregating each individual’s detected SVs that were singletons in each replicate after stratification (Fig. 3C). For LCL5, we observed that 54.01% (249 SVs) of replicate singleton SVs were shared between family members and 45.99% (212 SVs) were unique to LCL5. Similar proportions of each overlapped with the PacBio SV call set at 44.98% and 49.06%, respectively (Additional file 6: Table S6). Singleton replicate variability across family members did not show an enrichment of false positives or negative SVs based on this analysis.

Lastly, we investigated the impact of duplicate reads marking (dedup) and base quality score recalibration (recal) on the SV calling reproducibility. Differences between these steps per sample were the smallest contributor to variability after stratifying for variability from centers, mappers, and replicates. For LCL5, we defined a working set of 1653 SVs of which 150 were singletons and 1503 were non-singletons. 68% of singleton and 86.96% of non-singleton SVs overlapped with the PacBio call set. Thus, most of the variability in the call sets was likely due to false negative calls attributed to the differences between the dedup or recal call sets. For singleton SVs, we observed that 33.33% (50 SVs) overlapped with family singletons while 66.67% (100 SVs) did not (Fig. 3D). Of singleton SVs overlapping family singletons, 62% overlapped with the LCL5 PacBio call set while 71% of singleton SVs not overlapping family singletons overlapped with the PacBio call set. The majority of the LCL5 singleton SVs (80 SVs) were attributed to the dedup strategy while a minority (70 SVs) were part of the recal call set.

### Best practices for short-read-based SV calling

In the previous sections, we defined and ranked the analytically accessible sources of variability impacting the SV call sets. This included examining alignment methods, insert sizes/coverage, and variability attributed to centers and replicates. We further examined all the different short-read sequencing pipelines and results to identify the most robust settings and benchmarked its variability using the PacBio long-reads SV call set. We observed that Stampy consistently resulted in the highest number of SVs calls (~15,076.55 across LCL5), however, with the smallest ratio of 21.89% overlapping SVs (~3301.61 SVs across LCL5) with the Pacbio call set (Additional file 8: Table S7). In contrast, Bowtie2 resulted in the least SVs called (~7704.55 SVs across LCL5), but with a higher ratio of 32.83% overlapping SVs (2529.83 SVs on average) with the PacBio call set (Additional file 8: Table S7). For non-overlapping SV, we observed that the major difference was seen in an increase in duplications, inversions followed by insertion calls. For example, for LCL5, the portion of falsely detected SVs increases from Bowtie2 (~5174.72 SVs), BWA-MEM (~8370.67 SVs), and Isaac (~9308.78 SVs) to Stampy (~11774.94 SVs) in that order, respectively.

Next, we investigated if by taking a consensus between mappers the SV calling can be improved. We observed that using a consensus between mapping techniques increased precision (Additional file 8: Table S7). This is especially useful when utilizing SV mappers that have a high sensitivity such as Stampy and Isaac, but initially a lower precision compared to Bowtie2. For example, for LCL5, SVs identified by both alignments from Isaac and Stampy (~8384.83 SVs) had a 34.18% overlap with Pacbio (2866.72 SVs) compared to Isaac alone (26.57%) or Stampy alone (21.9%). Whereas an overlap between Bowtie2 and Stampy did not yield a high recall (~5190.33 SV with a recall of 15.66%) although it had higher precision (~2376.5 SVs or 45.79%).

In summary, we observed that Bowtie2 mappings lead to the SV sets with the highest precision, but lowest sensitivity. This might promote its usage in settings where precision is more important outside of the research setting or in the clinical setting. Nevertheless, Bowtie2 potentially leads to less precise breakpoints that could hinder the interpretation of the SV itself. Combined approaches using Stampy, Isaac, and BWA-MEM allow for a less precise albeit a more sensitive approach to detecting SVs, which lends itself better to research applications. These observed trends hold true across SV call sets from all family members across the different mappers (Additional file 8: Table S7).

## Discussion

In this study, we investigated the impact of factors that influenced our ability to accurately identify SVs. This is an important step towards the robust and routine identification of SVs in research and medical applications where SV calling is currently underrepresented. By investigating three replicates for each of the family members of a Chinese quartet, we were able to identify the source of variability between replicates and between sequencing centers. We showed that variability between sequencing centers appears to be the second largest source of variability, some of which could be explained by varying coverage between the centers. The SV mapping methods showed the largest impact likely due to the different heuristics and their ability to provide split read alignments. This of course also impacted the coverage slightly in certain regions, but not overall (Additional file 7: Fig. S2). The least impactful source of variability on SV calling was the base pair recalibration and marking of the duplicated reads. While this step is recommended for SNV calling (as part of the Genome Analysis Toolkits best practices), it did not have a significant impact on the SV analysis. Over the entire analysis, we did not identify any significant clustering of the highly variable events (Fig. 3E, Additional file 9: Table S8, and Additional file 10: Table S9). Moreover, we observed that a significant proportion of singletons for sequencing centers and replicates were indeed true positives and thus indicate a higher false negative (i.e., missing) rate by others. This concurs with previous literature highlighting a lower sensitivity over short-read approaches [12, 28]. On the other hand, we did not observe a high false positive rate per our analysis and filtering strategy given that we did not take translocations into account. These translocations have been previously discussed as large sources of false positive SV calls (e.g., representing repeat expansion) [2, 3, 28].

This study provides new insights for the discussion of reproducibility among NGS data sets. Previous studies showed a low reproducibility given the same data set due to incomplete workflow documentation or differences in versions of the methods used [29]. However, this can be overcome with documentation and careful analysis. What remains challenging, especially for SV identification, seems to be the heterogeneous study design especially across sequencing centers [30]. This is important for consideration of small to midsize projects, but unavoidable for large scale studies such as TOPMed etc. Thus, simple checks like the singleton rate per center, sequencing technology, or PCA would potentially highlight these issues together with SV genotyping of the identified variation [31]. Another strategy would be to use SV genotyping methods such as SVTyper [32] or Paragraph [33] to go back with a candidate list of SVs and check for any support across the samples. We applied this here for a subset of SV that showed the highest diversity (center and mapper only SV). We found that while the majority of LCL5 bam files are supportive of the deletions we genotyped there is still substantial variability present (see Additional file 7: Fig. S3). This indicates that the variability of SV is not due to individual callers but indeed is based much deeper as certain samples do not indicate a deletion even down to a single read support. Still SV genotyping remains challenging and thus certain SV were not genotyped at all [31].

Moreover, our study might have underestimated the variability of SV calling given our stringent SV calling pipeline. While this led to deeper insights into each of the sources of variability, we might have inadvertently filtered out some other specific variability since we could not fully independently investigate the impact from the sequencing center (e.g., coverage) vs. the mapping-based biases. Nevertheless, the aim of this study is not to obtain a comprehensive SV call set or another SV calling benchmark, but rather better understand underlying mechanisms. Lastly, we focused on only one family of Chinese ethnicity, however, the approach and comparisons were standardized steps independent of the sample origin and should thus reflect a similar trend across populations.

## Conclusions

This study is the first step to better understand the variability that is often observed for SV calling and its associated challenges. This work paves the way for new QC and SV calling methods that are likely needed to overcome many challenges. Improving short-read SV calling by using multiple mapping methods can have a profound impact on precision and sensitivity in SV calling. Consideration would still need to be given to the compute cost of such an approach. Thus, it may be more practical for smaller scale projects. Insights obtained here could lead to a more routine application of SV calling for humans and other organisms, which should allow for more insights into biology and medicine.

## Methods

### Ethics, consent, and sample ascertainment

This study was approved by an independent ethics committee at the Fudan University School of Life Sciences. A Chinese family quartet from the Fudan Taizhou cohort consisting of two identical twins and their biological parents were consented to participate in this research. Sample DNA was extracted from lymphoblastoid cell lines (LCLs) derived from Epstein-Barr Virus transformed B cells. Specifically, LCLs were maintained and sub-cultured every 3–4 days using RPMI 1640 (Gibico Catalog No. 31870-082) supplemented with 10% fetal bovine serum (Gibico 10091-148). Cells were cultured at 37^o^C with 5% CO_2_ for six passages (2×10^9^ cells) before total DNA extraction. Cells were washed with PBS twice before DNA extraction using a Blood and Cell Culture DNA Maxi Kit (QIAGEN 13362) and stored in TE buffer (10 mM TRIS, pH 8.0, 1 mM EDTA, pH 8.0).

### Next-generation sequencing

Libraries were prepared consistently across three sequencing centers (Annoroad (ARD), NovoGene (NVG) and WuXi NextCODE (WUX)) using 200 ng of DNA with the Illumina TruSeq DNA nano following manufacturer’s instructions. DNA fragmentation was achieved using a Covaris (LE220) instrument with a target size of 350bp. All libraries were assessed for quantity and quality using the Qubit 3.0 fluorometer with the Quant-iT dsDNA HS Assay kit (ThermoFisher Scientific, Q32854) and the Agilent 2100 Bioanalyzer or TapeStation instrument. All materials were prepared with three replicates in a single batch at each sequencing center. They were then sequenced on the Illumina X10 platform with paired end 150 bp read length leveraging synthesis (SBS) chemistry per the manufacturer’s instructions.

### Short-read SV identification

SVs were mapped to the human reference genome (GRCh38 with decoy sequences from NCI-GDC) using default settings for BWA-MEM (v0.7.15), ISAAC (v1.0.7), Stampy (v1.0.29), and Bowtie2 (v2.2.9). Duplicate read marking (dedup) and base pair recalibration (recal) were applied for each SV mapper call set (creating two independent SV call sets per SV mapper per sample set) followed by SV calling with MetaSV [25] and Parliament2 [26] pipelines. The Parliament2 (v0.1.8) (https://github.com/dnanexus/parliament2) SV calls were generated using the default setting. Results from DELLY [34], BreakDancer [35], LUMPY [36], Manta [37], and CNVnator [38] were used as inputs into Parliament2 [26]. The MetaSV call sets were generated using MetaSV [25] (v0.5.4) (https://github.com/bioinform/metasv) with default settings. Specifically, special options were ‘--boost_sc --disable_assembly --max_ins_cov_frac 2 --min_support_frac_ins 0.015 --min_support_ins 25 --max_ins_intervals 10000 --age_window 50 --extraction_max_read_pairs 20000 --min_inv_subalign_len 100 --min_del_subalign_len 100 --svs_to_softclip INS INV DEL DUP --svs_to_report INV DEL INS DUP --svs_to_assemble INV DEL DUP INS’. Results from BreakSeq [39], BreakDancer [35], Pindel [40], and CNVnator [38] were used as inputs into MetaSV. For Parliament2, we filtered based on the recommendation: Include Manta only calls and otherwise only calls that are supported by at least two SV callers. For MetaSV, the recommendation was to filter for PASS only variations. Subsequently, the so filtered calls were merged using SURVIVOR [4] with the following parameters for all merges: a maximum distance of 1000 bp measured pairwise from the beginning and ends of each SV, respectively, SVs were required to be the same type and larger than 30bp. Per sample, a union set of MetaSV and Parliament2 results were generated. This resulted in the generation of 288 total SV call sets for downstream analyses (4 samples x 4 mappers x 3 centers x 3 replicates x 2 dedup/recal).

### Mapper comparisons

The SURVIVOR package was used to merge VCF files with the following parameters for all merges: a maximum distance of 1000 bp measured pairwise from the beginning and end of each SV respectively, SVs were required to be the same type, and SVs had to be larger than 30 bp. Overlapping SVs were stratified for dedup/recal variability using a SURVIVOR merge per sample, replicate, mapper, and center with a requirement of 2 out of 2 dedup and recal supporting calls. The SVs were then stratified for replicate variability using a SURVIVOR merge per sample, mapper, and center with a requirement of 3 out of 3 replicates supporting an SV. Center variability was stratified for using a SURVIVOR merge per sample and mapper with a requirement of 3 out of 3 centers supporting an SV. A union merge using SURVIVOR per sample was then used to combine SVs from the 4 different mappers for downstream analyses with a minimum SV size of 50 bp.

### Center comparisons

The SURVIVOR package was used to merge VCF files with the following parameters for all merges: a maximum distance of 1000 bp measured pairwise from the beginning and ends of each SV, respectively, SVs were required to be the same type, and larger than 30 bp. Overlapping SVs were stratified for dedup/recal variability using a SURVIVOR merge per sample, replicate, mapper, and center with a requirement of 2 out of 2 dedup and recal supporting calls. Mapper variability was stratified for using a SURVIVOR merge per sample, replicate, and center with a requirement of 4 out of 4 mappers supporting an SV. Replicate variability was stratified for using a SURVIVOR merge per sample and center with a requirement of 3 of 3 replicates supporting an SV. A union merge using SURVIVOR per sample was then used to combine SVs from the different centers for downstream analyses with a minimum SV size of 50 bp.

### Replicate comparisons

The SURVIVOR package was used to merge VCF files with the following parameters for all merges: a maximum distance of 1000 bp measured pairwise from the beginning and ends of each SV respectively, SVs were required to be the same type, and larger than 30 bp. Overlapping SVs were stratified for dedup/recal variability using a SURVIVOR merge per sample, replicate, mapper, and center with a requirement of 2 out of 2 dedup and recal supporting calls. Mapper variability was stratified for using a SURVIVOR merge per sample, replicate and center with a requirement of 4 out of 4 mappers supporting an SV. Center variability was stratified for using a SURVIVOR merge per sample and replicate with a requirement of 3 out of 3 centers supporting an SV. A union merge using SURVIVOR per sample was then used to combine SVs for the different replicates for downstream analyses with a minimum SV size of 50 bp.

### Dedup/recalibration comparisons

The SURVIVOR package was used to merge VCF files with the following parameters for all merges: a maximum distance of 1000 bp measured pairwise from the beginning and ends of each SV respectively, SVs were required to be the same type and larger than 30 bp. SVs were stratified for mapper variability using SURVIVOR merges per sample, center, and replicate with a requirement of 4 out of 4 mappers supporting an SV. The SVs were then stratified for replicate variability using a SURVIVOR merge per sample and center with a requirement of 3 out of 3 replicates supporting an SV. Center variability was stratified for using a SURVIVOR merge per sample with a requirement of 3 out of 3 centers supporting an SV. A union merge using SURVIVOR per sample was then used to combine SVs from the dedup and recal pipelines for downstream analyses with a minimum SV size of 50 bp.

### GIAB high confidence regions filter

We obtained GIAB v0.6 high confidence regions (ftp://ftp-trace.ncbi.nlm.nih.gov/giab/ftp/data/AshkenazimTrio/analysis/NIST_SVs_Integration_v0.6/HG002_SVs_Tier1_v0.6.bed ) [12] and used NCBI (NCBI Genome Remapping Service) to map these coordinates from hg19 to GRCH38. SV call sets were subsequently filtered with the aforementioned regions using bedtools intersect before they were compared across family members and strategies.

### Generation and comparisons to long-read-based SV calls

Samples LCL5-8 were sequenced utilizing the Pacific Biosciences (PacBio) platform. Specifically, PacBio SMRTbell libraries (CLR = Continuous Long Read) were constructed with the standard PacBio library preparation protocols using 20 kb insert size preparation solution, and the sequencing was conducted on PacBio Sequel (Pacific Biosciences, USA) platform.

The main steps for library preparation are: (1) gDNA shearing, (2) DNA damage repair, (3) blunt end-ligation with hairpin adapters from the SMRTbell Express Template Prep Kit 2.0 (Pacific Biosciences, USA), (4) size selection, and (5) binding to polymerase. A total amount of 2 μg DNA per sample was used for the DNA library preparations. The genomic DNA sample was sheared by g-TUBEs (Covaris, USA) according to the expected size of the fragments for the library. Single-strand overhangs were then removed and DNA fragments were damage-repaired, end-polished, and ligated with the stem-loop adaptor for PacBio sequencing. Link-failed fragments were further removed by exonuclease, and target fragments were screened by the BluePippin (Sage Science, USA). The SMRTbell library was then purified using AMPure PB and Agilent 2100 Bioanalyzer (Agilent technologies, USA) was used to detect the size of Library fragments. Reads were mapped to GRCH38 using minimap2 (v 2.17-r941) [41]. Subsequent SVs were identified using Sniffles (v1.0.11) [28] with default parameters. SV calls from different analysis strategies were then merged with the PacBio SV call set for comparison using SURVIVOR with the following parameters: a maximum distance of 1000 bp measured pairwise from the beginning and ends of each SV, respectively, SVs were required to be the same type and larger than 50bp.

### Coverage and insert size analysis

For coverage analysis, mean coverage over a window size of 1KB was computed using samtools. For each sample, the mean and standard deviation of coverage were analyzed against the number of detected SVs. Similarly, for each sample, the mean and standard deviation of insert size were analyzed against the number of detected SVs.

### Cross mapper comparisons

The SURVIVOR package was used to merge all VCF files per sample (LCL5-8). The following parameters were used for all merges: a maximum distance of 1000 bp measured pairwise from the beginning and ends of each SV, respectively, SVs were required to be the same type, and larger than 50 bp. A resulting VCF file was then used per sample (encompassing all mapper, center, technical replicates, and dedup/recal SV call sets) to calculate total SVs resulting from each mapper SV call set. Additionally, comparisons were done across mappers using the same VCF file as input.

### SV genotyping

SVTyper (version 0.7.1) was run on two sets of SV (center specific and mapper specific variability) with the default parameters on all LCL5 bam files. Subsequently, we filtered the VCF file and kept only those SVs that had at least a single read support. These SV were then merged using SURVIVOR with a 1000bp distance and requiring the same SV type. A custom perl script was used to extract the SUPP_VEC values reported by SURVIVOR for each SV. R was used to plot the histograms per input VCF file to represent the overall agreement across all different bam files for LCL5.

## Supplementary Information

**Additional file 1: Table S1.** General statistics for the LCL5 sample set pre-filtering requiring an overlap between MetaSV and Parliament2 call sets.

**Additional file 2: Table S2.** Total and average number of SVs with a genic or intergenic only impact.

**Additional file 3: Table S3.** Distribution of total and singleton SV types per strategy.

**Additional file 4: Table S4.** Distribution of total and singleton SV types per strategy by SV size.

**Additional file 5: Table S5.** Comparison of variability from centers, replicates, mappers and dedup/recal after stratifying for other contributors.

**Additional file 6: Table S6.** Comparison of Overlap for LCL5 singleton SVs, family singleton SVs and LCL5 PacBio.

**Additional file 7: Figure S1.** Comparison of the distribution of singleton SVs across family members per center per sample. **Figure S2.** Comparison of the distribution of mean coverage by different SV mappers (Bowtie2, BWA-MEM, Isaac and Stampy) for LCL5. **Figure S3.** Examining evidence for variable singleton SVs from center (A) and mapper (B) using SVTyper.

**Additional file 8: Table S7.** Distribution of SVs prefiltering by mapper, overlapping across mapper call sets and benchmarked with the long-read PacBio SV call set.

**Additional file 9: Table S8.** Distribution of singletons SVs across strategies (mapper, replicate, center, dedup/recal) for LCL5 in 100,000 and 1,000,000 base pair windows.

**Additional file 10: Table S9.** Distribution of LCL5 singleton SVs across chromosomes for all strategies (mapper, replicate, center, dedup/recal).

## Data Availability

The raw sequencing data that support the findings of this study are restricted, but are available from the SEQC2 study group upon request. Other supporting data are available in the Additional files.
