# Supplementary material for: Hidden biases in germline structural variant detection
Source: Genome Biol. 2021 Dec 20;22:347. doi: 10.1186/s13059-021-02558-x (PMC8686633; doi:10.1186/s13059-021-02558-x)
Supplement: Supplementary file 7 — Additional file 7: Figure S1. Comparison of the distribution of singleton SVs across family members per center per sample. Figure S2. Comparison of the distribution of mean coverage by different SV mappers (Bowtie2, BWA-MEM, Isaac and Stampy) for LCL5. Figure S3. Examining evidence for variable singleton SVs from center (A) and mapper (B) using SVTyper. [file 13059_2021_2558_MOESM7_ESM.docx]

*
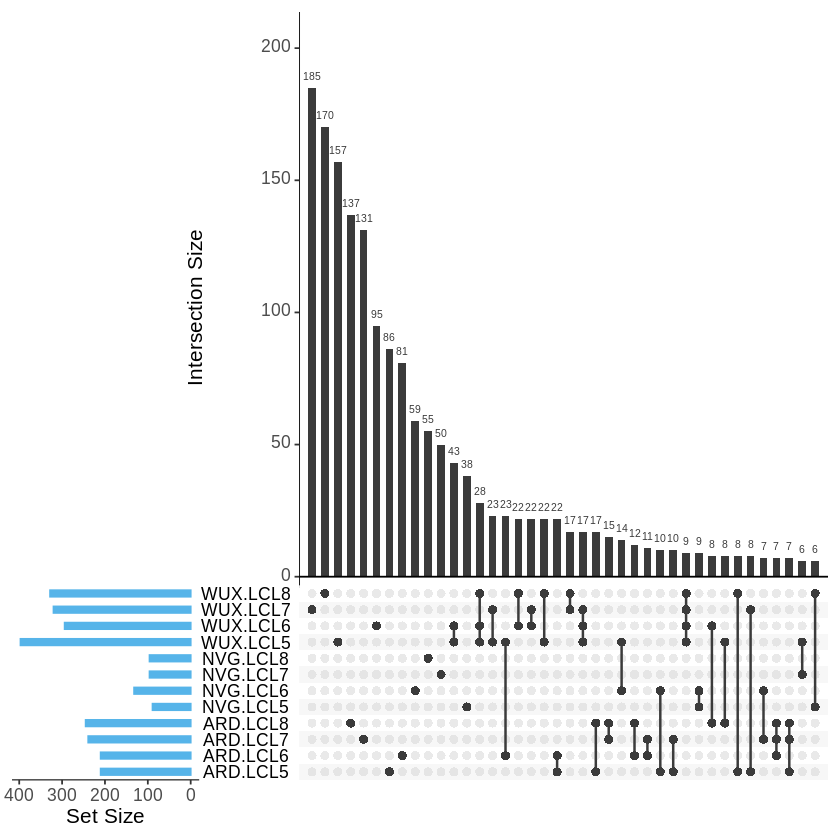
*

***Figure S1:*** *Comparison of the distribution of singleton SVs across family members per center per sample*


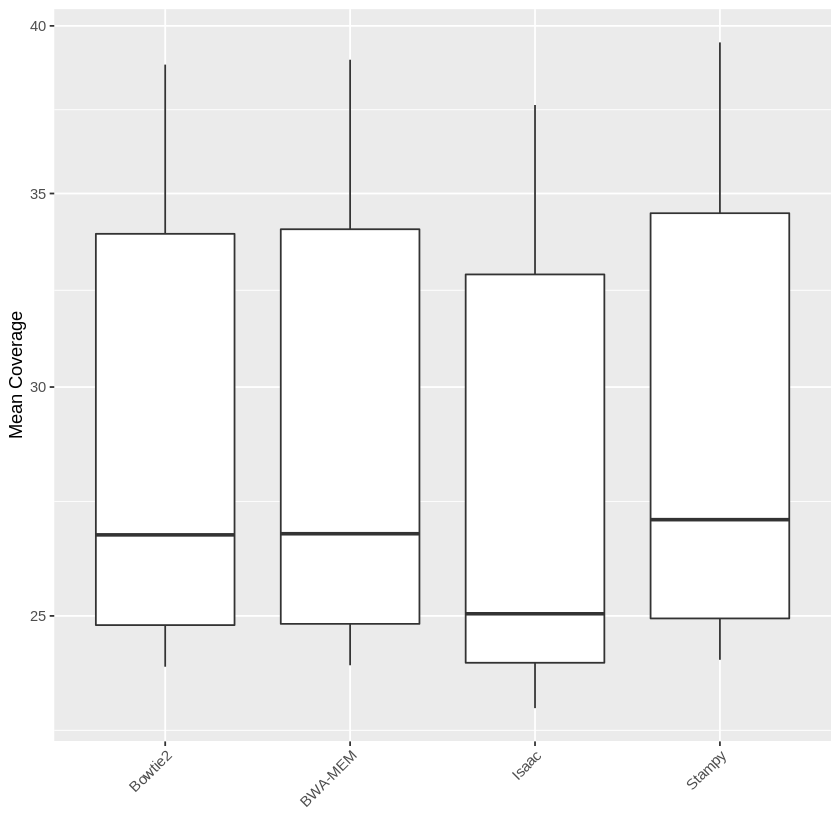


***Figure S2:*** *Comparison of the distribution of mean coverage by different aligners (Bowtie2, BWA-MEM, Isaac and Stampy) for LCL5*


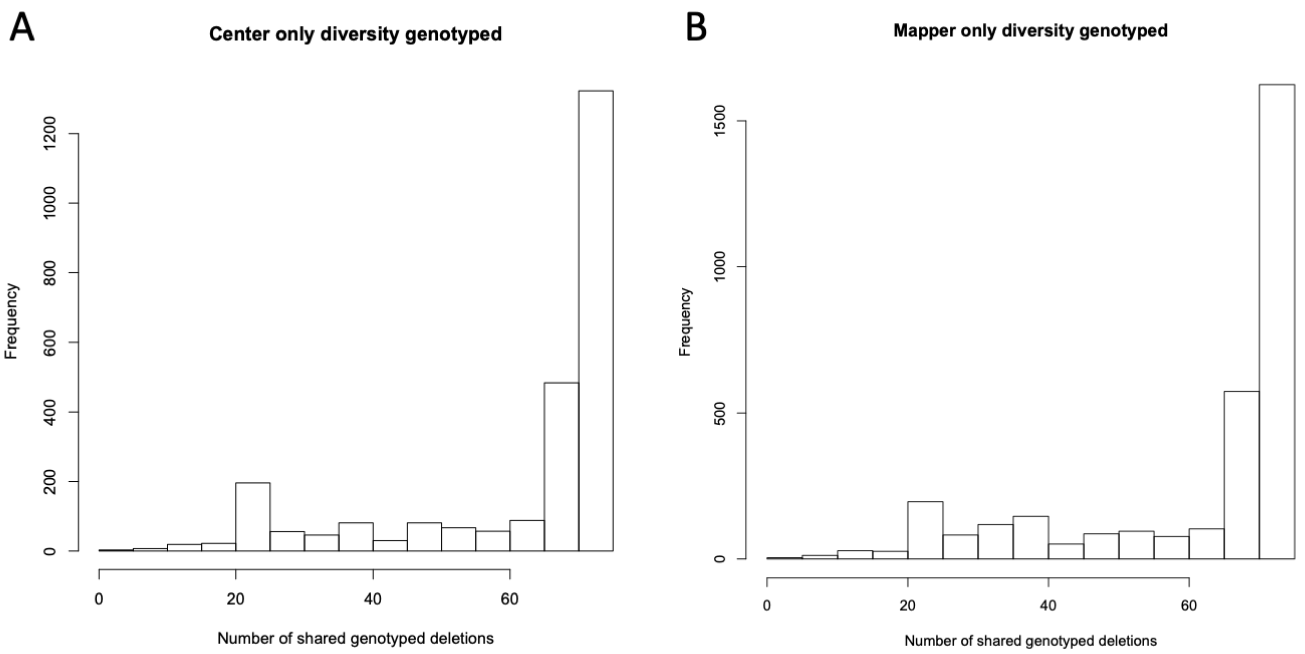


***Figure S3:*** *Examining evidence for variable singleton SVs from center (A) and mapper (B) using SVTyper*
